# Extracellular Ca^2+^ modulates ADP-evoked aggregation through altered agonist degradation: implications for conditions used to study P2Y receptor activation

**DOI:** 10.1111/j.1365-2141.2010.08499.x

**Published:** 2011-04

**Authors:** Sarah Jones, Richard J Evans, Martyn P Mahaut-Smith

**Affiliations:** Department of Cell Physiology and Pharmacology, University of LeicesterLeicester, UK

**Keywords:** platelets, aggregation, calcium, ectonucleotidases, ADP

## Abstract

ADP is considered a weak platelet agonist due to the limited aggregation responses it induces *in vitro* at physiological concentrations of extracellular Ca^2+^ [(Ca^2+^)_o_]. Lowering [Ca^2+^]_o_ paradoxically enhances ADP-evoked aggregation, an effect that has been attributed to enhanced thromboxane A_2_ production. This study examined the role of ectonucleotidases in the [Ca^2+^]_o_-dependence of platelet activation. Reducing [Ca^2+^]_o_ from millimolar to micromolar levels converted ADP (10 μmol/l)-evoked platelet aggregation from a transient to a sustained response in both platelet-rich plasma and washed suspensions. Blocking thromboxane A_2_ production with aspirin had no effect on this [Ca^2+^]_o_-dependence. Prevention of ADP degradation abolished the differences between low and physiological [Ca^2+^]_o_ resulting in a robust and sustained aggregation in both conditions. Measurements of extracellular ADP revealed reduced degradation in both plasma and apyrase-containing saline at micromolar compared to millimolar [Ca^2+^]_o_. As reported previously, thromboxane A_2_ generation was enhanced at low [Ca^2+^]_o_, however this was independent of ectonucleotidase activity_._ P2Y receptor antagonists cangrelor and MRS2179 demonstrated the necessity of P2Y_12_ receptors for sustained ADP-evoked aggregation, with a minor role for P2Y_1_. In conclusion, Ca^2+^-dependent ectonucleotidase activity is a major factor determining the extent of platelet aggregation to ADP and must be controlled for in studies of P2Y receptor activation.

ADP is an important platelet agonist during haemostasis and thrombosis, exerting its effects through two G-protein-coupled receptors P2Y_1_ and P2Y_12_ (Gachet, 2008). P2Y_1_ is coupled to G_αq_, leading to an increase in cytosolic calcium through stimulation of phospholipase C_β_ (PLC_β_) (Jin *et al*, 1998; Savi *et al*, 1998; Leon *et al.* 1999), whereas P2Y_12_ is coupled to G_αi_, leading to activation of phosphatidylinositol 3-Kinase (PI3-K) (Trumel *et al*, 1999; Jackson *et al*, 2005) and inhibition of adenylate cyclase. For platelet-platelet adhesion, and thus thrombus formation, activation of the fibrinogen receptor α_IIb_β_3_ is required, a process that depends upon concomitant stimulation of G_αq_ and G_αi_ signalling pathways (Jin & Kunapuli, 1998). Several platelet agonists bind to receptors coupled to G_αq_, including thrombin, thromboxane A_2_ (TXA_2_) and ADP. However, ADP is the only platelet agonist that stimulates G_αi_ signalling at physiological concentrations and is therefore an essential co-stimulus to achieve full functional responses for all known platelet agonists (Dorsam & Kunapuli, 2004; Gachet, 2008). Despite the central role of ADP in platelet aggregation and thrombogenesis, it is normally considered to be a weak platelet agonist due to the reversible nature of the aggregation response observed *in vitro* at physiological levels of external Ca^2+^ (Gachet, 2008). Extremely low levels of extracellular Ca^2+^ abolish fibrinogen binding to α_IIb_β_3_ integrin, however at micromolar extracellular calcium concentrations, ADP-evoked aggregation is enhanced compared to physiological Ca^2+^ levels and not readily reversible (Mustard *et al*, 1975; Packham *et al*, 1989). Although this paradoxical effect was reported more than two decades ago, the underlying basis whereby extracellular calcium modulates ADP-evoked aggregation remains unclear. It has been proposed that millimolar Ca^2+^ levels inhibit TXA_2_ generation via altered ERK phosphorylation (Garcia *et al*, 2007), leading to loss of secondary aggregation (Mustard *et al*, 1975; Packham *et al*, 1989), however exactly how Ca^2+^ achieves this effect is not known.

Following stimulation of platelet P2Y receptors with ADP, the duration and amplitude of the response can be regulated by two principal mechanisms, firstly, desensitization of the P2Y receptors preventing further signalling and, secondly, removal of ADP by ectonucleotidases. Ectonucleotidases comprise a large family of extracellular nucleotide degrading enzymes including ectonucleoside triphosphate diphosphohydrolases (E-NTPDases), ectonucleotide pyrophosphatase/phosphodiesterases (E-NPPs), alkaline phosphatases and 5′ nucleotidase (Zimmermann, 2000). ADP derived from platelets and other blood cells is thought to predominantly be metabolised by E-NTPDase1 (CD39), a membrane-bound enzyme expressed by endothelial cells, lymphocytes and macrophages (Kansas *et al*, 1991; Marcus *et al*, 1997), as well as microparticles that originate from these cell types (Atkinson *et al*, 2006; Banz *et al*, 2008). CD39 converts ADP to AMP, which is subsequently converted to adenosine, an inhibitor of platelet function, by 5′ nucleotidase (CD73) expressed on endothelial cells and in plasma (Coade & Pearson, 1989; Zimmermann, 2000; Heptinstall *et al*, 2005). There is also evidence that soluble E-NPPases in plasma can degrade ADP directly to adenosine (Birk *et al*, 2002; Cauwenberghs *et al*, 2006), thus ectonucleotidases can convert prothrombotic mediators into inhibitors of platelet activation.

In this study we have investigated further the mechanism(s) underlying the differential responses to ADP at physiological compared to low (micromolar) extracellular calcium concentrations. We demonstrate that degradation of ADP by Ca^2+^-dependent ectonucleotidases is an important factor in determining the amplitude and duration of platelet aggregation. The results have consequences for understanding the effectiveness of ADP as a platelet agonist, and in the selection of experimental conditions to explore P2Y receptor activation.

## Materials and methods

### Materials

CHRONO-LUME was purchased from Labmedics (Manchester, UK), Pyruvate kinase was obtained from Roche Diagnostics Limited (East Sussex, UK), GF109203X and MRS2179 were purchased from Tocris (Bristol, UK). Thromboxane B_2_ (TXB_2_) assay kits were purchased from Cambridge Bioscience LTD (Cambridge, UK). Cangrelor (ARC-69931MX) was a kind gift from AstraZeneca (Moindal, Sweden). ADP, apyrase (gradeVII), aspirin, phosphoenolpyruvate and all other chemicals were purchased from Sigma (Poole, UK).

### Platelet preparation

Blood was obtained from healthy, aspirin-free, volunteers according to a protocol approved by the local ethical committee of the University of Leicester. Blood was drawn from the forearm by venepuncture into a syringe containing acid citrate dextrose anticoagulant (ACD: 85 mmol/l trisodium citrate, 78 mmol/l citric acid, 111 mmol/l glucose) 9:1 *v/v*. Platelet-rich plasma (PRP) was obtained by centrifugation at 700 ***g*** for 5 min. When re-calcified, 20 mmol/l CaCl_2_ [calculated using a Nomogram (Hastings *et al*, 1934)] was added to citrated PRP to achieve [Ca^2+^]_o_ of approximately 2 mmol/l immediately prior to each experiment. The extracellular Ca^2+^ in nominally Ca^2+^-free saline and in similar citrated plasma:saline mixtures has been estimated to be approximately 20 and 17 μmol/l respectively (Packham *et al*, 1987; Rolf *et al*, 2001).

To prepare washed platelet suspensions, apyrase (0·32 u/ml) and, where stated, aspirin (100 μmol/l or 1 mmol/l) were added to the PRP and platelets pelleted by centrifugation at 350 ***g*** for 20 min. Platelets were then resuspended in a volume of nominally Ca^2+^-free saline (145 mmol/l NaCl, 5 mmol/l KCl, 1 mmol/l MgCl_2_ 10 mmol/l HEPES, 10 mmol/l glucose, 1 g/l fibrinogen pH 7·35) equal to that of the removed plasma, with or without apyrase (0·32 u/ml) as required by the specific experiment. In experiments performed at physiological calcium concentrations, 2 mmol/l CaCl_2_ was added to the platelets immediately prior to use.

### Platelet aggregation

PRP or washed platelet suspensions were diluted (1:1) in saline with or without apyrase (0·32 u/ml) and stimulated with ADP at 37°C under stirring conditions. Aggregation was measured using optical aggregometry (Model 400 lumi-aggregometer; Chronolog, Havertown, PA, USA).

### Platelet disaggregation

Washed, apyrase-free platelets were stimulated with ADP (10 μmol/l) at 37°C under stirring conditions in the presence of 2 mmol/l Ca^2+^. After 2 min, apyrase (0·32 u/ml), the P2Y_1_ receptor antagonist MRS2179 (10 μmol/l), the P2Y_12_ receptor antagonist AR-C69931MX (1 μmol/l) or a saline control was added to the suspension. Disaggregation was assessed 3 min after the addition of the P2Y receptor antagonists or apyrase and calculated as a percentage of the peak ADP-evoked aggregation.

### ADP measurement

The concentration of extracellular ADP was assessed by luciferin:luciferase luminescence measurements after conversion to ATP via a method adapted from Heath (2004). Briefly, 2 min after addition of 10 μmol/l ADP to plasma or apyrase-containing saline, with or without Ca^2+^, 50 μl samples were removed and added to a mixture of 420 μl Tris-K acetate buffer (100 mmol/l Tris-acetate, 2 mmol/l EDTA, 25 mmol/l potassium acetate), 10 μl pyruvate kinase/phosphoenolpyruvate (prepared by mixing equal volumes of 10 mg/ml pyruvate kinase and 200 mmol/l phosphoenolpyruvate) and 20 μl CHRONO-LUME. Luminescence was measured using a Model 400 lumi-aggregometer (Chronolog) and converted to ATP levels based upon a calibration curve for each batch of CHRONO-LUME.

### TXB_2_ measurements

TXB_2_ synthesis was measured as an indication of TXA_2_ production due to the highly labile nature of TXA_2_. Washed platelets were stimulated with ADP (10 μmol/l) at 37°C under stirring conditions for 3 min in the presence and absence of apyrase (0·32 u/ml), in both physiological Ca^2+^ and nominally Ca^2+^-free conditions, and reactions terminated by snap freezing. For analysis of TXB_2_, samples were thawed and centrifuged at 3000 ***g*** for 10 min at 4°C. The supernatant was diluted 1:5 using the buffer supplied with the assay kit and TXB_2_ determined according to the manufacturer's instructions (Cambridge Bioscience).

### Statistics

Records of aggregation are from individual experiments, typical of 3–7 donors. Differences between means ± SEM were assessed using paired Student's *t*-test and a *P* value of <0·05 was considered to be significant. *P* values are indicated at levels of <0·05 (*), <0·01 (**) and <0·001 (***).

## Results

### Extracellular Ca^2+^ levels regulate ADP-evoked aggregation independently of TXA_2_ synthesis

**Fig 1:**
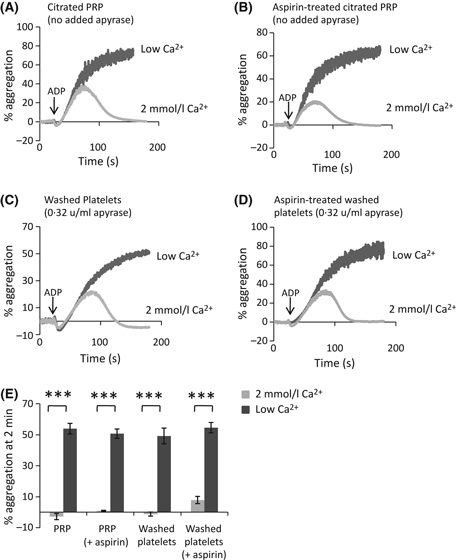
Platelet responses to ADP are sustained at low extracellular calcium concentrations. Sample (A–D) or average (E) responses to 10 μmol/l ADP without added extracellular Ca^2+^ (low Ca^2+^) or in the presence of approximately 2 mmol/l external Ca^2+^. (A) citrated PRP; (B) citrated PRP treated with aspirin (100 μmol/l); (C) platelets resuspended in saline containing apyrase (0·32 u/ml); (D) platelets resuspended in saline containing apyrase (0·32 u/ml) and aspirin (100 μmol/l); (E) Aggregation measured at 2 min.

ADP (10 μmol/l) evoked a sustained aggregation of platelets in plasma anti-coagulated with citrate that reduced the extracellular Ca^2+^ concentration [(Ca^2+^)_o_] to the micromolar range (Fig 1A, E; average peak aggregation of 53·9 ± 3·4%). When the medium was recalcified to approximately 2 mmol/l free Ca^2+^, the aggregation was converted to a transient response that returned to baseline levels of transmission (−2·8 ± 2%) within approximately 2 min (Fig 1A, E). Previous studies of this [Ca^2+^]_o_-dependent aggregation response showed that production of TXA_2_ was enhanced at micromolar compared to millimolar [Ca^2+^]_o_ levels and concluded that secondary stimulation of TXA_2_ receptors is responsible for the reversible nature of the ADP-evoked aggregation (Mustard *et al*, 1975; Packham *et al*, 1989; Garcia *et al*, 2007). However, we observed a similar effect of [Ca^2+^]_o_ on aggregation when TXA_2_ synthesis was blocked by aspirin (Fig 1B, E; average values at 2 min of 50·7 ± 3% and 0·9 ± 0·3% in low and physiological Ca^2+^ levels respectively). In platelets resuspended in a physiological saline with apyrase, 10 μmol/l ADP evoked a transient aggregation response in the presence of 2 mmol/l [Ca^2+^]_o_, which was also converted to a sustained response by omission of CaCl_2_ from the saline (Fig 1C, E; transmission levels 2 min after ADP of 0·6 ± 2% and 53·2 ± 5·4%, respectively), in agreement with reports by other groups (Mustard *et al*, 1975; Packham *et al*, 1989). As observed for platelets in the presence of plasma, aspirin did not block the sustained aggregation in salines with micromolar [Ca^2+^]_o_ (Fig 1D, E; average values at 2 min of 55·9 ± 1·3% and 7·1 ± 4·2% in low and physiological Ca^2+^ levels respectively). To ensure that TXA_2_ generation was completely inhibited, experiments were repeated in the presence of 1 mmol/l aspirin, which also had no significant effect on the ability of reduced [Ca^2+^]_o_ to enhance platelet aggregation (*P* > 0·05, data not shown). Together, these data suggest that factor(s) other than altered TXA_2_ production must contribute to the ability of reduced [Ca^2+^]_o_ to enhance ADP-evoked aggregation.

### Prevention of ADP degradation abolishes the reversal of aggregation by calcium

**Fig 2:**
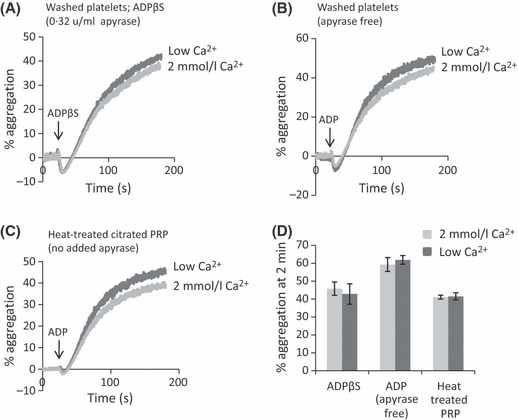
Prevention of ADP degradation leads to sustained aggregation at physiological extracellular calcium concentrations. Sample (A–C) or average (D) aggregation responses without added extracellular Ca^2+^ (low Ca^2+^) or in the presence of approximately 2 mmol/l external Ca^2+^. (A) Washed platelets (containing 0·32 u/ml apyrase) stimulated with the hydrolysis-resistant analogue ADPβS (10 μmol/l); (B) platelets soon after resuspension in apyrase-free saline stimulated with ADP (10 μmol/l); (C) platelets resuspended in heat-treated citrated plasma stimulated with ADP (10 μmol/l); (D) Average aggregation measured at 2 min.

Given that our washed platelet preparation contained apyrase (E-NTPDase1 isolated from potato to prevent P2Y receptor desensitization) and PRP has been reported to contain endogenous ectonucleotidases, we considered whether degradation of ADP contributed to the transient nature of the ADP-evoked aggregation at millimolar Ca^2+^ levels. Aggregation evoked by the hydrolysis-resistant analogue ADPβS was not significantly different in the presence or absence of extracellular Ca^2+^ (Fig 2A, D; aggregation at 2 min of 45·8 ± 3·7% and 42·9 ± 5·6% in normal and low [Ca^2+^]_o_, respectively; *P* > 0·05). Moreover, when platelets were resuspended in the absence of apyrase, and experiments performed rapidly to limit the effects of desensitization, ADP-evoked aggregation was also sustained in the presence of 2 mmol/l extracellular Ca^2+^ (Fig 2B, D; 59·3 ± 3·8 and 61·9 ± 2·4% in normal and low Ca^2+^ respectively). Finally, the [Ca^2+^]_o_-dependence of ADP-evoked aggregation responses in plasma was abolished when platelets were resuspended in autologous heat-treated plasma (60°C, 30 min) to destroy enzymatic activity (Fig 2C, D; 41·1 ± 1·1 and 41·5 ± 1·9% in millimolar *versus* micromolar [Ca^2+^]_o_ respectively). Together, these observations are consistent with a role for Ca^2+^-dependent nucleotidase activity in the [Ca^2+^]_o_-dependence to ADP-evoked sustained aggregation responses.

### ADP degradation is accelerated by millimolar calcium concentrations

**Fig 3:**
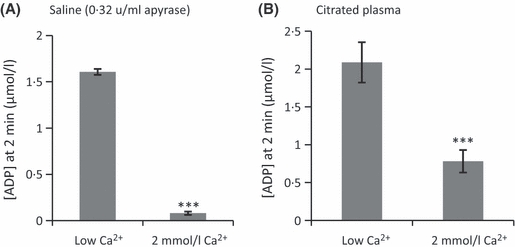
ADP degradation by apyrase and endogenous ectonucleotidases present in plasma is reduced at micromolar calcium concentrations. Degradation of a single bolus of 10 μmol/l ADP was assessed by measurements of the concentration of ADP remaining after 2 min in (A) saline containing apyrase (0·32 u/ml) in the nominal absence of Ca^2+^ or in the presence of 2 mmol/l Ca^2+^; and (B) citrated plasma before and after re-calcification to 2 mmol/l.

To directly assess the extent of ADP degradation in millimolar *versus* micromolar [Ca^2+^]_o_ concentrations, ADP (10 μmol/l) was added to apyrase-treated saline or platelet-free plasma and the ADP concentration after 2 min measured by luminescence following conversion to ATP (see *Methods*). The concentration of ADP remaining in nominally Ca^2+^-free saline was 1·61 ± 0·06 μmol/l, which was significantly reduced to 0·079 ± 0·03 μmol/l (*P* < 0·001) in the presence of 2 mmol/l Ca^2+^, indicating accelerated nucleotidase activity by physiological [Ca^2+^]_o_ (Fig 3A). Similarly, ADP incubated with citrated plasma was degraded, from 10 to 2·1 ± 0·27 μmol/l, by enzymes endogenous to plasma, whereas under recalcified conditions the ADP remaining was markedly lower, at 0·78 ± 0·14 μmol/l (*P* < 0·001) (Fig 3B). A significant effect of [Ca^2+^]_o_ on degradation of 10 μmol/l ADP was also detected at earlier time points, as shown by measurements after only 10 s (Figure S1). This suggests that platelets in the presence of millimolar Ca^2+^ are exposed to a reduced level of ADP throughout most of the experiment compared to at micromolar Ca^2+^ levels. In contrast, addition of 2 mmol/l MgCl_2_ to nominally Ca^2+^-free saline did not significantly affect ADP degradation or lead to a transient aggregation response (Figure S2). Together with the data in Fig 2, these results support the conclusion that reduced ADP degradation substantially contributes to the paradoxical amplifying effect of reducing Ca^2+^ on platelet aggregation. This is also consistent with the reported enhancement of ectonucleotidase activity at millimolar concentrations of calcium compared to its nominal absence or in the presence of a chelator, such as EGTA (Christoforidis *et al*, 1995; Strobel *et al*, 1996; Marcus *et al*, 1997).

### Reversal of aggregation is due to removal of ADP, not negative feedback by adenosine

**Fig 4:**
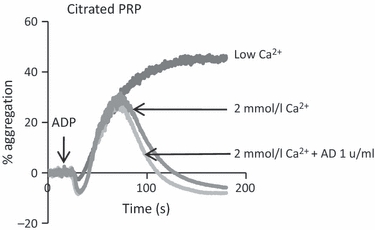
Transient ADP-evoked aggregation responses resulting from Ca^2+^-dependent ectonucleotidase activity do not involve negative feedback by adenosine. Platelet aggregation stimulated by ADP (10 μmol/l) in nominally Ca^2+^-free conditions or in the presence of 2 mmol/l Ca^2+^ with or without adenosine deaminase (AD, 1 u/ml).

In plasma, 5′ nucleotidases convert AMP generated by the degradation of ATP and ADP to adenosine (Coade & Pearson, 1989; Heptinstall *et al*, 2005), thus we also considered whether the transient responses to ADP in plasma involved inhibition via G_αs_-coupled adenosine A2a receptors. In citrated PRP, adenosine (10 μmol/l) inhibited ADP (10 μmol/l)-evoked responses, resulting in aggregation responses similar to those observed with ADP in physiological calcium concentrations (Figure S3). This inhibition by adenosine was abolished by the addition of adenosine deaminase (1 u/ml). In recalcified PRP, the addition of adenosine deaminase had no effect on ADP-evoked aggregation (Fig 4), indicating that negative feedback by the generation of adenosine does not contribute to the reversibility of ADP-mediated responses.

### Sustained aggregation to ADP requires constant P2Y_12_ receptor stimulation

**Fig 5:**
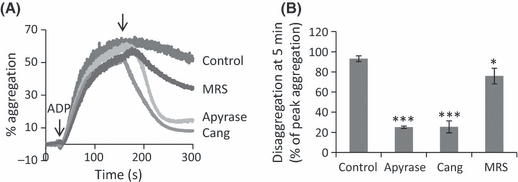
Reversal of aggregation by ADP degrading enzymes is largely due to the termination of P2Y_12_ receptor signalling. (A) Aggregation of washed platelets (apyrase-free) was stimulated with ADP (10 μmol/l) soon after resuspension in the presence of 2 mmol/l Ca^2+^ and after 2 min (arrow) one of the following was added: apyrase (0·32 u/ml), ARC-69931MX (Cang, 1 μmol/l), MRS2179 (MRS, 10 μmol/l), or a vehicle control. (B) Disaggregation was measured 3 min after the addition of the inhibitors or vehicle and calculated as a percentage of peak ADP-evoked aggregation response.

We next sought to determine whether reversal of aggregation by Ca^2+^-dependent nucleotidases was due to loss of activation of P2Y_1_, P2Y_12_ or both receptors. Disaggregation of ADP (10 μmol/l)-stimulated platelets was measured in response to apyrase (0·32 u/ml), the P2Y_12_ antagonist cangrelor (1 μmol/l) or the P2Y_1_ antagonist MRS2179 (10 μmol/l) (Fig 5A). Cangrelor reversed aggregation by 74·6 ± 5·9%, comparable to that observed with apyrase 74·9 ± 1·2% (Fig 5B). In contrast, MRS2179 caused a more moderate reversal of aggregation (reduction of 24·0 ± 7·8%; Fig 5B). Thus, reversal of ADP-induced aggregation by ectonucleotidases is largely due to loss of signalling through P2Y_12_ receptors.

### TXA_2_ generation is enhanced at low extracellular calcium concentrations independently of altered ectonucleotidase activity

**Fig 6:**
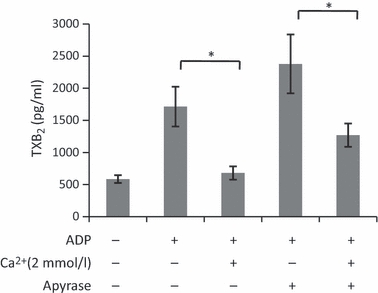
TXA_2_ generation is enhanced at low extracellular calcium concentrations independently of apyrase activity. ADP (10 μmol/l)-evoked thromboxane B_2_ production (as a measure of TXA_2_ generation) in washed suspensions of platelets with and without apyrase in nominally Ca^2+^-free conditions or in the presence of 2 mmol/l extracellular Ca^2+^. TXB_2_ was measured 3 min after addition of ADP.

To investigate whether the reduced TXA_2_ generation previously reported by others (Harfenist *et al*, 1987; Packham *et al*, 1987, 1989) at millimolar [Ca^2+^]_o_ was a consequence of termination of ADP signalling by ectonucleotidases, the effect of apyrase (0·32 u/ml) at micromolar and millimolar [Ca^2+^]_o_ was examined on TXB_2_ production from washed platelets 3 min after stimulation with 10 μmol/l ADP (Fig 6). As reported previously (Harfenist *et al*, 1987; Packham *et al*, 1987, 1989), TXB_2_ production from apyrase-treated platelets was markedly reduced at physiological extracellular Ca^2+^ concentrations compared to that observed in the nominal absence of Ca^2+^. However, similar results were observed in saline lacking apyrase. This indicates that although increased [Ca^2+^]_o_ reduces TXB_2_ synthesis, this is not dependent on the effect of Ca^2+^ on ectonucleotidases.

### Relative contribution of ADP degradation *versus* P2Y receptor desensitization in limiting platelet responses to ADP

**Fig 7:**
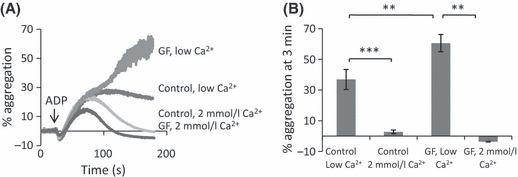
ADP degradation is the predominant mechanism regulating ADP-mediated platelet aggregation at physiological extracellular Ca^2+^ concentrations. (A) Platelet aggregation evoked by 2 μmol/l ADP in PRP before and after re-calcification, in the presence and absence of the PKC inhibitor GF 109203X (10 μmol/l). (B) Average aggregation 3 min after addition of ADP.

P2Y_1_ and P2Y_12_ receptors are both susceptible to receptor desensitization after prolonged agonist stimulation (Hardy *et al*, 2005; Mundell *et al*, 2006). To determine the relative importance of receptor desensitization *versus* ectonucleotidase activity in ADP-evoked aggregation, the pan-PKC inhibitor GF109203X was used to attenuate receptor desensitization and aggregation was measured in citrated PRP before and after recalcification (Fig 7A). An intermediate concentration of ADP (2 μmol/l) was used for these experiments to unmask a clear potentiating effect of PKC inhibition, which increased the sustained aggregation at 3 min from 36·9 ± 6·5% to 60·5 ± 5·6% in micromolar [Ca^2+^]_o_ (Fig 7B). In contrast, after recalcification, aggregation in the presence of GF109203X or vehicle control, was not significantly different and returned to baseline levels of −3·5 ± 1·6% and 2·7 ± 1·2% (*P* > 0·05), respectively, 3 min after stimulation (Fig 7B). Thus, ADP degradation overrides any contribution by P2Y receptor desensitization to the reversal of aggregation in physiological external Ca^2+^ concentrations.

## Discussion

Reports of differential platelet responses to ADP in physiological *versus* nominally Ca^2+^-free conditions emerged over 20 years ago (Mustard *et al*, 1975; Packham *et al*, 1989). These studies concluded that enhanced TXA_2_ production accounts for the paradoxical amplifying effect of lowering Ca^2+^ on ADP-evoked aggregation. The present study now shows that altered degradation of ADP can also contribute to this phenomenon. The known Ca^2+^-dependence of ecto-ADPases (Marcus *et al*, 1997; Zimmermann, 2000) provides the basis for the difference observed in millimolar *versus* micromolar [Ca^2+^]_o_ and this conclusion is supported by direct measurements of ADP. The sustained aggregation evoked by ADP is largely due to stimulation of P2Y_12_ receptors, consistent with previous reports of the more crucial role of this G_i_-coupled pathway compared to P2Y_1_ in amplifying responses to ADP, collagen and thrombin receptors (Trumel *et al*, 1999; Dorsam & Kunapuli, 2004; Hechler *et al*, 2005; Jackson *et al*, 2005; Cosemans *et al*, 2006). The present results also highlight the importance of controlling for nucleotide breakdown in studies of P2 receptor signalling when the external Ca^2+^ concentration is modified. For example, it is common practice to include soluble apyrase to limit P2 receptor desensitization within *in vitro* experiments and simply to vary the external [Ca^2+^] to investigate the relative contribution of Ca^2+^ entry *versus* release pathways in nucleotide-evoked signalling events.

The limited aggregation response at normal [Ca^2+^]_o_ has contributed to the view that ADP is a ‘weak platelet agonist’. However, when metabolism of ADP is limited, the ability of this agonist to stimulate sustained aggregation, as shown in the present study, is more consistent with the substantial reduction in platelet activation observed in P2Y_1_ and P2Y_12_ receptor-deficient mice (Fabre *et al*, 1999; Leon *et al*, 1999; Andre *et al*, 2003) and the major role of ADP in amplifying collagen- and thrombin-evoked responses *in vitro*. It is possible that dense granule secretion evoked by collagen and thrombin provides a more sustained source of ADP compared to the single bolus application used in standard *in vitro* experiments. Furthermore, ATP (and thus also ADP) remains sustained for a considerable time near sites of vascular injury (Born & Kratzer, 1984), probably reflecting the continual recruitment and activation of platelets during the haemostatic process. Thus, the *in vitro* experimental condition that limits ADP degradation, such as micromolar [Ca^2+^]_o_, may more closely represent the ability of this agonist to stimulate platelet function *in vivo*. Alternatively, use of a non-hydrolysable analogue, such as ADPβS, or repeated application of ADP to replace degraded agonist should be considered within *in vitro* studies designed to investigate mechanisms of ADP-dependent platelet activation.

Whilst ADP degradation significantly contributed to the transient nature of the responses to ADP in millimolar [Ca^2+^]_o_, we agree with earlier studies (Packham *et al*, 1989; Garcia *et al*, 2007) that TXA_2_ generation is lower at millimolar compared to micromolar [Ca^2+^]_o_. In our experiments, the marked enhancement of TXB_2_ generation by lowering [Ca^2+^]_o_ was also observed in apyrase-free saline, indicating that the Ca^2+^-dependent modulation of TXA_2_ generation can occur independently of effects on ectonucleotidase activity. It has been reported that reduced TXA_2_ synthesis in physiological calcium concentrations is the result of inhibited ERK phosphorylation (Garcia *et al*, 2007), however the process by which this is achieved is unclear, and whether these effects are downstream of an extracellular event or whether calcium influx is required, remains to be investigated. In the present study, 10 μmol/l ADP stimulated sustained aggregation at millimolar [Ca^2+^]_o_ in the absence of apyrase, conditions under which there was no detectable TXA_2_ generation, suggesting that this response is independent of secondary signalling through TXA_2_ receptors. At lower concentrations of ADP, however, the release of secondary agonists is required to achieve full aggregation, therefore the effect of extracellular calcium on TXA_2_ production may be more significant, and modulation of ADP-evoked aggregation by [Ca^2+^]_o_ may result from a combination of both altered ectonucleotidase activity and TXA_2_ production.

Although we did not observe any difference in aggregation within the normal physiological range of extracellular calcium concentrations (0·5–2 mmol/l) (three donors, data not shown), results from this study demonstrate the impact of variable ectonucleotidase activity on platelet function, which may have profound implications in certain clinical conditions. It has previously been reported that in blood from patients with elevated leucocyte counts, degradation of ADP is accelerated and aggregation in response to ADP is reduced due to increased NTPDase levels (Pulte *et al*, 2007; Glenn *et al*, 2008). Moreover, in a rat model of cholestatic liver disease where plasma ectonucleotidase activity is enhanced, reduced aggregation was exhibited in response to ADP and low dose collagen (which is dependent on ADP secretion) (Witters *et al*, 2010). Conversely, individuals demonstrating reduced ectonucleotidase expression may have more reactive platelets and be more susceptible to thrombotic events. Such patients may benefit from therapeutic intervention with soluble forms of NTPDase1.

In conclusion, the present study shows that reduced degradation of ADP by ectonucleotidases contributes to the paradoxical amplification of ADP-evoked aggregation at micromolar compared to millimolar extracellular Ca^2+^ levels. The sustained inside-out activation of fibrinogen receptors that occurs in response to ADP at low [Ca^2+^]_o_ is likely to be more representative of the potential contribution of ADP to a developing thrombus *in vivo*, where a constant supply of this P2Y receptor agonist from activated platelets can override enzymatic clearance in the vicinity of a developing thrombus.

## Supporting Information

**Fig S1.** Reduced ADP degradation in low (micromolar) calcium concentrations is evident after 10 s. (A) Degradation of ADP (10 μmol/l) by apyrase (0.32 u/ml) in nominally Ca^2+^ free conditions or in the presence of 2 mmol/l Ca^2+^ measured 10 s after ADP addition. (B) Degradation of ADP (10 μmol/l) by ectonucleotidases present in plasma was measured before and after re-calcification, 10 s after ADP addition.

**Fig S2.** Elevated extracellular Mg^2+^ does not accelerate ADP degradation. (A) Washed platelets stimulated with ADP (10 μmol/l) in nominally Ca^2+^-free saline, or following addition of 2 mmol/l Mg^2+^. (b) Degradation of ADP (10 μmol/l) by apyrase (0.32 u/ml) present in the platelet saline buffer measured after 2 min in nominally Ca^2+^-free saline or following addition of 2 mmol/l Mg^2+^.

**Fig S3.** Adenosine deaminase blocks the inhibition of ADP-mediated aggregation by adenosine. Aggregation of washed platelets stimulated with ADP (10 μmol/l) in the presence of adenosine (10 μmol/l) with or without prior addition of adenosine deaminase (AD) 1 u/ml.

## References

[b1] Andre P, Delaney SM, LaRocca T, Vincent D, DeGuzman F, Jurek M, Koller B, Phillips DR, Conley PB (2003). P2Y12 regulates platelet adhesion/activation, thrombus growth, and thrombus stability in injured arteries. Journal of Clinical Investigation.

[b2] Atkinson B, Dwyer K, Enjyoji K, Robson SC (2006). Ecto-nucleotidases of the CD39/NTPDase family modulate platelet activation and thrombus formation: potential as therapeutic targets. Blood Cells, Molecules, and Diseases.

[b3] Banz Y, Beldi G, Wu Y, Atkinson B, Usheva A, Robson SC (2008). CD39 is incorporated into plasma microparticles where it maintains functional properties and impacts endothelial activation. British Journal of Haematology.

[b4] Birk AV, Bubman D, Broekman MJ, Robertson HD, Drosopoulos JH, Marcus AJ, Szeto HH (2002). Role of a novel soluble nucleotide phospho-hydrolase from sheep plasma in inhibition of platelet reactivity: hemostasis, thrombosis, and vascular biology. Journal of Laboratory and Clinical Medicine.

[b5] Born GV, Kratzer MA (1984). Source and concentration of extracellular adenosine triphosphate during haemostasis in rats, rabbits and man. Journal of Physiology.

[b6] Cauwenberghs S, Feijge MA, Hageman G, Hoylaerts M, Akkerman JW, Curvers J, Heemskerk JW (2006). Plasma ectonucleotidases prevent desensitization of purinergic receptors in stored platelets: importance for platelet activity during thrombus formation. Transfusion.

[b7] Christoforidis S, Papamarcaki T, Galaris D, Kellner R, Tsolas O (1995). Purification and properties of human placental ATP diphosphohydrolase. European Journal of Biochemistry.

[b8] Coade SB, Pearson JD (1989). Metabolism of adenine nucleotides in human blood. Circulation Research.

[b9] Cosemans JM, Munnix IC, Wetzker R, Heller R, Jackson SP, Heemskerk JW (2006). Continuous signaling via PI3K isoforms beta and gamma is required for platelet ADP receptor function in dynamic thrombus stabilization. Blood.

[b10] Dorsam RT, Kunapuli SP (2004). Central role of the P2Y12 receptor in platelet activation. Journal of Clinical Investigation.

[b11] Fabre JE, Nguyen M, Latour A, Keifer JA, Audoly LP, Coffman TM, Koller BH (1999). Decreased platelet aggregation, increased bleeding time and resistance to thromboembolism in P2Y1-deficient mice. Nature Medicine.

[b12] Gachet C (2008). P2 receptors, platelet function and pharmacological implications. Thrombosis and Haemostasis.

[b13] Garcia A, Shankar H, Murugappan S, Kim S, Kunapuli SP (2007). Regulation and functional consequences of ADP receptor-mediated ERK2 activation in platelets. Biochemical Journal.

[b14] Glenn JR, White AE, Johnson AJ, Fox SC, Myers B, Heptinstall S (2008). Raised levels of CD39 in leucocytosis result in marked inhibition of ADP-induced platelet aggregation via rapid ADP hydrolysis. Platelets.

[b15] Hardy AR, Conley PB, Luo J, Benovic JL, Poole AW, Mundell SJ (2005). P2Y1 and P2Y12 receptors for ADP desensitize by distinct kinase-dependent mechanisms. Blood.

[b16] Harfenist EJ, Packham MA, Kinlough-Rathbone RL, Cattaneo M, Mustard JF (1987). Effect of calcium ion concentration on the ability of fibrinogen and von Willebrand factor to support the ADP-induced aggregation of human platelets. Blood.

[b17] Hastings AB, Mclean F, Eichelberger L, Lowell Hall J, Da Costa E (1934). The ionization of calcium, magnesium, and strong citrates. The Journal of Biological Chemistry.

[b18] Heath MF (2004). Secretion from dense granules: luminescence method for adenine nucleotides. Methods in Molecular Biology.

[b19] Hechler B, Cattaneo M, Gachet C (2005). The P2 receptors in platelet function. Seminars in Thrombosis and Hemostasis.

[b20] Heptinstall S, Johnson A, Glenn JR, White AE (2005). Adenine nucleotide metabolism in human blood – important roles for leukocytes and erythrocytes. Journal of Thrombosis and Haemostasis.

[b21] Jackson SP, Schoenwaelder SM, Goncalves I, Nesbitt WS, Yap CL, Wright CE, Kenche V, Anderson KE, Dopheide SM, Yuan Y, Sturgeon SA, Prabaharan H, Thompson PE, Smith GD, Shepherd PR, Daniele N, Kulkarni S, Abbott B, Saylik D, Jones C, Lu L, Giuliano S, Hughan SC, Angus JA, Robertson AD, Salem HH (2005). PI 3-kinase p110beta: a new target for antithrombotic therapy. Nature Medicine.

[b22] Jin J, Kunapuli SP (1998). Coactivation of two different G protein-coupled receptors is essential for ADP-induced platelet aggregation. Proceedings of the National Academy of Sciences of the USA.

[b23] Jin J, Daniel JL, Kunapuli SP (1998). Molecular basis for ADP-induced platelet activation. II. The P2Y1 receptor mediates ADP-induced intracellular calcium mobilization and shape change in platelets. The Journal of Biological Chemistry.

[b24] Kansas GS, Wood GS, Tedder TF (1991). Expression, distribution, and biochemistry of human CD39. Role in activation-associated homotypic adhesion of lymphocytes. Journal of Immunology.

[b25] Leon C, Hechler B, Freund M, Eckly A, Vial C, Ohlmann P, Dierich A, LeMeur M, Cazenave JP, Gachet C (1999). Defective platelet aggregation and increased resistance to thrombosis in purinergic P2Y1 receptor-null mice. Journal of Clinical Investigation.

[b26] Marcus AJ, Broekman MJ, Drosopoulos JH, Islam N, Alyonycheva TN, Safier LB, Hajjar KA, Posnett DN, Schoenborn MA, Schooley KA, Gayle RB, Maliszewski CR (1997). The endothelial cell ecto-ADPase responsible for inhibition of platelet function is CD39. Journal of Clinical Investigation.

[b27] Mundell SJ, Jones ML, Hardy AR, Barton JF, Beaucourt SM, Conley PB, Poole AW (2006). Distinct roles for protein kinase C isoforms in regulating platelet purinergic receptor function. Molecular Pharmacology.

[b28] Mustard JF, Perry DW, Kinlough-Rathbone RL, Packham MA (1975). Factors responsible for ADP-induced release reaction of human platelets. American Journal of Physiology.

[b29] Packham MA, Kinlough-Rathbone RL, Mustard JF (1987). Thromboxane A2 causes feedback amplification involving extensive thromboxane A2 formation on close contact of human platelets in media with a low concentration of ionized calcium. Blood.

[b30] Packham MA, Bryant NL, Guccione MA, Kinlough-Rathbone RL, Mustard JF (1989). Effect of the concentration of Ca^2+^ in the suspending medium on the responses of human and rabbit platelets to aggregating agents. Thrombosis and Haemostasis.

[b31] Pulte D, Olson KE, Broekman MJ, Islam N, Ballard HS, Furman RR, Olson AE, Marcus AJ (2007). CD39 activity correlates with stage and inhibits platelet reactivity in chronic lymphocytic leukemia. Journal of Translational Medicine.

[b32] Rolf MG, Brearley CA, Mahaut-Smith MP (2001). Platelet shape change evoked by selective activation of P2X1 purinoceptors with alpha, beta-methylene ATP. Thrombosis and Haemostasis.

[b33] Savi P, Beauverger P, Labouret C, Delfaud M, Salel V, Kaghad M, Herbert JM (1998). Role of P2Y1 purinoceptor in ADP-induced platelet activation. FEBS Letters.

[b34] Strobel RS, Nagy AK, Knowles AF, Buegel J, Rosenberg MD (1996). Chicken oviductal ecto-ATP-diphosphohydrolase. Purification and characterization. The Journal of Biological Chemistry.

[b35] Trumel C, Payrastre B, Plantavid M, Hechler B, Viala C, Presek P, Martinson EA, Cazenave JP, Chap H, Gachet C (1999). A key role of adenosine diphosphate in the irreversible platelet aggregation induced by the PAR1-activating peptide through the late activation of phosphoinositide 3-kinase. Blood.

[b36] Witters P, Hoylaerts M, Freson K, de Vos R, van Pelt J, Nevens F, van Geet C, Cassiman D (2010). ADP-degrading enzymes inhibit platelet activation in bile duct-ligated rats. Journal of Thrombosis and Haemostasis.

[b37] Zimmermann H (2000). Extracellular metabolism of ATP and other nucleotides. Naunyn-Schmiedebergs Archives of Pharmacology.

