# Supplementary figures and images for: Extracellular Ca2+ modulates ADP-evoked aggregation through altered agonist degradation: implications for conditions used to study P2Y receptor activation
Source: Br J Haematol. 2011 Apr;153(1):83–91. doi: 10.1111/j.1365-2141.2010.08499.x (PMC3084511; doi:10.1111/j.1365-2141.2010.08499.x)

**A)**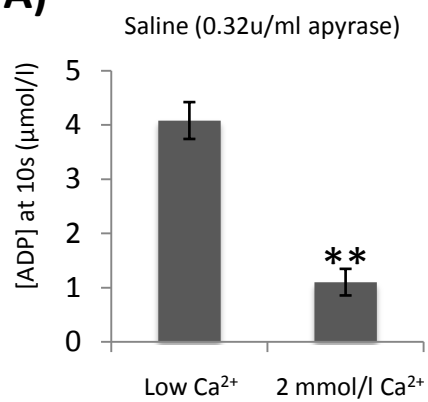**B)**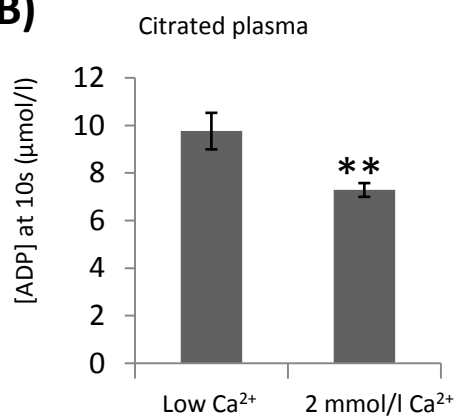

Supplementary Figure 1.

Supplement: Supplementary file 1 [file bjh0153-0083-SD1.pdf]

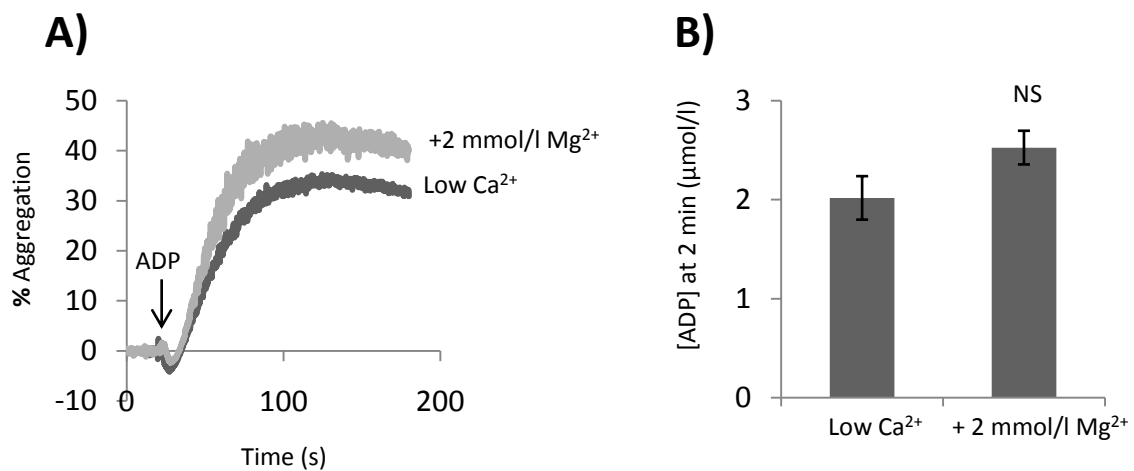

Supplementary Figure 2.

Supplement: Supplementary file 2 [file bjh0153-0083-SD2.pdf]

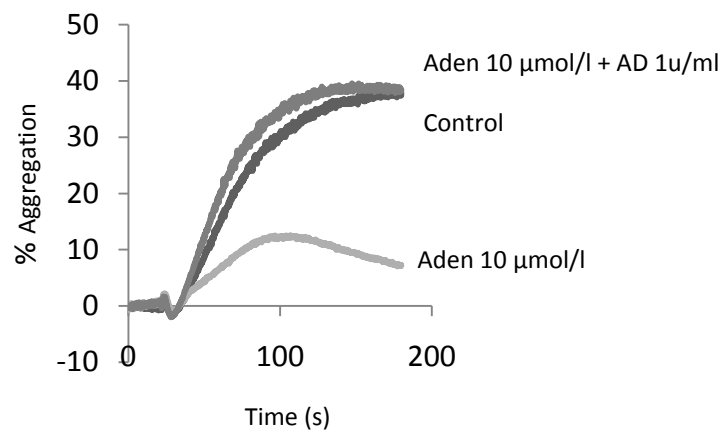

Supplementary Figure 3.

Supplement: Supplementary file 3 [file bjh0153-0083-SD3.pdf]
